# The long-term effects of parental marriage age on children’s educational human capital

**DOI:** 10.1371/journal.pone.0322151

**Published:** 2025-05-07

**Authors:** Bohui Yuan, Yanping Pu

**Affiliations:** School of Public Policy and Administration, Chongqing University, China; IIPS: International Institute for Population Sciences, INDIA

## Abstract

This paper focuses on micro-level Chinese households and studies the impact of parental marriage age on the education outcomes of their children, utilizing data from the China Family Panel Studies (2020). Through OLS model regression, it is found that parents who get married later are better prepared to have children, marital stability is also stronger, and their children have a longer education period. But this positive effect will turn negative after the age of marriage exceeds 30. Further analysis reveals that parental age at marriage has a heterogeneous effect on their children’s educational attainment, which means mothers have a more significant impact on their children’s education outcomes than fathers.

## 1. Introduction

Enhancing children’s human capital is pivotal for improving the transmission of intergenerational poverty and fostering social equity. The disparity in familial investment, often called the intergenerational investment gap, is the principal factor contributing to the variance in personal human capital. The existing research on the influencing factors of family investment in children’s human capital has shifted from economic factors such as parent’s education, income, and type of work[1] to the number of children’s siblings, childhood left behind or migration experience, parents’ marital quality and mothers’ marriage age in recent years [2–4]. Marriage establishes the smallest unit responsible for child-rearing as a social mechanism for determining parents for children, leading to the reorganization and development of resources within two families. Different marriage patterns and parental characteristics are crucial in children’s human capital accumulation. Mothers’ delay in marriage has a positive impact on children’s human capital by promoting their educational acquisition, matching higher-quality spouses, and improving marital stability [5–6].

However, on the one hand, the existing literature focuses on examining the relationship between women’s marriage age and children’s education and health status. There are few studies on the influence of parents’ marriage age on children’s education and most of them ignore the differential impact of fathers’ and mothers’ marriage age. On the other hand, previous studies on education and health human capital focus on the short-term and linear effects, measured mainly by children’s reading, numeracy, and academic performance, and pay little attention to the long-term and nonlinear results of intergenerational human capital investment.

This study measures the results of intergenerational human capital investment based on education after children complete school. It considers the long-term impact of the marriage age of fathers and mothers on children’s human capital investment, which is helpful to enrich and expand the research content of the intergenerational transmission of educational human capital. Since the 21st century, the change in attitudes toward marriage and extending educational years has delayed the marriage age. As the marriage age is delayed, older parents become more mature psychologically and physically, they are better prepared to have children. Furthermore, compared with fathers, mothers spend more time caring for their children, which may significantly impact children’s education.

Based on the data of CFPS (2020), this study finds that the later the parents get married, the higher the education their children obtain. However, this positive effect will turn negative after the age of marriage exceeds 30, which means there is an inverted U-shaped relationship between the parental marriage age and the accumulation of children’s human capital. Furthermore, the impact of parents’ marriage age on their children’s education is different between fathers and mothers. This article provides causal evidence for parents’ marriage age to affect their children’s long-term education outcomes. It is beneficial to comprehend how social and economic inequalities are perpetuated or improved across generations. This knowledge is crucial for designing equitable educational policies that level the playing field for children from various family backgrounds.

## 2. Theoretical framework and research hypotheses

### 2.1 The nonlinear effect of parental marriage age on children’s human capital

Mill (1917) [7] posited that if a child’s early comprehension and memory abilities are comparable to those of peers, their academic achievements largely depend on family education. As the primary agents of family education, parents’ differential investments in their children’s human capital shape academic performance and have long-term implications for the quality of their children’s human capital accumulation [8]. This study examines how parental marriage age affects children’s educational human capital from two perspectives: direct effects and indirect effects.

#### 2.1.1 Direct effects: Intergenerational human capital investment.

From the perspective of life cycle theory, delaying marriage provides individuals with a critical time window to complete educational attainment (prolonging schooling), establish career trajectories (accumulating professional capital), and achieve psychological maturity (enhancing non-cognitive skills). This maturation effect directly influences intergenerational human capital investment, shaping children’s educational outcomes.

Life cycle theory conceptualizes life as a continuous developmental process in which individuals assume different social roles and face distinct developmental tasks at each stage [9–10]. Early marriage often coincides with critical periods of academic and career development, during which economic instability limits parents’ ability to invest in their children’s education and health [11–14]. Additionally, parents in the early stages of their careers tend to prioritize work commitments, reducing their availability for childcare and educational engagement. Young parenthood, mainly when unplanned or occurring outside of marriage, can introduce an external shock to family planning, further constraining the resources available for child development [15–16]. Under such circumstances, parenting often arises from external pressures rather than intrinsic motivation, which may diminish the intentionality and effectiveness of educational investment.

In contrast, delaying marriage allows individuals to extend their years of education [6,17] and transition into a phase of career stability [18]). Marriage at a more mature stage provides a stable social role and emotional support network, which broadens individuals’ perspectives and enhances professional prospects, ultimately leading to higher household incomes [19]. When material capital is sufficient, parents who marry later can allocate more significant financial resources toward high-quality educational investments, including conducive learning environments, educational expenditures, and nutritional support. Furthermore, greater psychological maturity enhances parents’ awareness of the long-term value of education, prompting them to adopt a strategic and sustained approach to educational planning [20–23]. For these parents, childbearing tends to be a deliberate decision embedded in long-term life planning [24], optimizing intergenerational human capital investment throughout the child’s life cycle.

However, excessively delayed marriage may also impose constraints on children’s educational outcomes. First, although late-marrying parents may be well-prepared for parenthood, their advanced career commitments could limit the time and energy available for child-rearing and academic involvement, potentially impeding children’s cognitive and non-cognitive skill development [25–26]. Second, intergenerational age misalignment may introduce financial and caregiving pressures, particularly as older parents enter retirement while their children are still in school. This dynamic may lead children to forgo higher education in favor of early labor market entry, thereby constraining their human capital accumulation. Third, more significant generational gaps between parents and children could impede effective communication and intergenerational understanding, weakening the educational environment [27–28].

These arguments suggest that parental marriage age exhibits a non-monotonic effect on children’s educational human capital, initially promoting and subsequently inhibiting educational outcomes as marriage age increases.

#### 2.1.2 Indirect effects: Marital stability.

Extensive research has established that marital stability is a critical determinant of child development [29–30], and declining marital stability can negatively impact children’s educational human capital accumulation [31]. Marital instability disrupts the family environment and fosters economic insecurity, elevating psychological stress among children and adolescents, potentially compromising their educational attainment [32–35].

Parental marriage age is closely linked to marital stability, yet this relationship is not linear. Early marriage is associated with an increased risk of divorce, as insufficient premarital information and emotional immaturity contribute to marital instability [36–38]. However, delayed marriage does not unconditionally enhance marital stability. Recent studies indicate that beyond a certain threshold, further increases in marriage age elevate the risk of divorce, as individuals may face heightened social pressure to marry “less compatible” or “suboptimal” partners [36,39,40].

This marginal effect suggests a non-linear relationship between marriage age and marital stability, indicating a potential U-shaped link to children’s human capital [41]. Within a moderate range, delaying marriage enhances marital resilience, leading to a more stable environment for child-rearing [42]. Stable marital unions facilitate more outstanding parental commitment to intergenerational human capital investment, yielding more decisive arithmetic and literacy outcomes in children [5]. However, excessive delays in marriage may introduce new sources of marital strain, ultimately undermining the benefits of late marriage for children’s educational development.

Drawing on these theoretical insights, this study proposes the following hypothesis:

**Hypothesis 1:** An inverted U-shaped relationship exists between parental marriage age and children’s educational human capital; intergenerational human capital investment and marital stability serve as key mechanisms.

### 2.2 The influence of parental marriage age on children’s education: A gendered perspective

Considering the interactive effects of spousal role allocation, parenting styles, and resource investment, this study further examines the differential impact of parental marriage age on children’s educational outcomes from four key perspectives:

First, mothers’ early engagement in childcare and maturity advantage. Due to biological factors, mothers typically assume childcare responsibilities during pregnancy, while fathers do not fully take their parental role until after the child’s birth. This results in a longer “lag period” for fathers. Even when parents marry at the same age, mothers may exhibit greater maturity than fathers, allowing them to play a more significant role in early childcare and educational decision-making.

Second, more excellent maternal time investment in childcare. Influenced by the traditional Chinese notion of “men as breadwinners and women as homemakers” and the associated household division of labor, married women often reduce their market labor participation and devote more time to child-rearing [43]. Bianchi (2000) [42] found that mothers spend approximately twice as much time on childcare as fathers. This suggests that maternal maturity may have a more pronounced effect on children’s human capital accumulation under the same marriage age conditions.

Third, delayed marriage enhances women’s economic capacity and decision-making power. Early marriage often shortens women’s educational attainment, limiting career development and leading to lower earnings compared to their later-marrying counterparts. In contrast, delayed marriage enables women to accumulate higher levels of human capital, thereby increasing their bargaining power and control over household resources [44]. This enhanced decision-making power significantly affects household investments, particularly in children’s education [5]. Studies have shown that improvements in maternal status within the household are often accompanied by increased spending on education and nutrition, as well as more incredible psychological support for children [45]. As a result, later-marrying women have a stronger willingness and a greater capacity to invest in their children’s human capital.

Fourth, asymmetric spousal influence in parenting decisions. Belsky & Steinberg (1979) [46] argue that wives influence their husbands more in childcare decisions than in the reverse. Consequently, given her dominant role in family decision-making, a more mature mother will likely have a more significant positive impact on children’s human capital accumulation.

Based on the above analysis, this study extends Hypothesis 1 by exploring the gender-specific effects of parental marriage age on children’s education and proposes the following hypothesis:

**Hypothesis 2:** Compared to paternal marriage age, an appropriate delay in maternal marriage age has a more substantial positive effect on intergenerational human capital accumulation.

**Fig 1:**
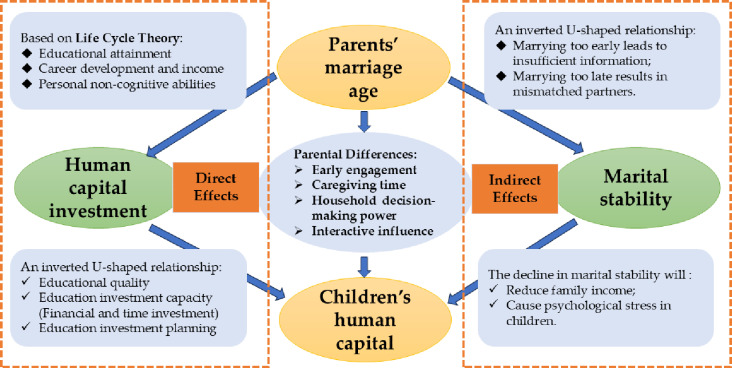
The impact of parental marriage age on children’s human capital. (A) Direct effects of parental marriage age on children’s human capital investment, illustrating an inverted U-shaped relationship where early marriage limits educational quality and investment capacity, while late marriage reduces partner compatibility. Key influencing factors include educational attainment, career development, and non-cognitive abilities. (B) Indirect effects mediated by marital stability: reduced stability lowers family income and increases psychological stress, which negatively affects human capital outcomes. (C) The effects of parental marriage age on children’s education vary through four channels: early engagement, caregiving time, household decision-making power, and interactive influence. Legend. The inverted U-shaped relationship indicates that a variable first increases and then decreases as another variable increases. “Investment capacity” refers to financial or time resources allocated to education, while “marital stability” reflects the longevity of the union and levels of conflict. Arrows indicate interactions between key variables.

Fig 1 presents the theoretical framework illustrating Hypothesis 1 and Hypothesis 2.

## 3. Materials and methods

### 3.1 Model

To investigate the influence of parents’ marriage age on their children’s educational human capital, the formula for the model is as follows:


$${Edu}_{i}=\alpha_{0}+\alpha_{1}{Marri}_{i}+\alpha_{2}{Marri}_{i}^{2}+\alpha_{3}X_{i}+\varepsilon_{i}$$ (1)

Among them ${Edu}_{i}\;$ represents the education of the i-th child; ${Marri}_{j}\;$ and ${Marri}_{i}^{2}\;$ represents the parents’ marriage age and the square of marriage age separately. We divided the square of marriage age by 100 in descriptive analysis and regressions to improve the readability of the results. $X_{i}$ refers to the characteristic variable of parents, family, and children, including essential characteristics such as parents’ years of education, health status, number of children, and gender.$\;\varepsilon_{i}$refers to the random error term.

### 3.2 Data source and processing

The data in this article mainly comes from the CFPS (2020) database of the China Family Panel Studies. The database takes individuals and families as the survey subject and contains information on three dimensions: community, family, and individual. Regarding the research questions in this study, the database can comprehensively reflect personal marriage, health, and education. Firstly, it should be noted that according to official information from CFPS, the investigation period began in early July 2020 and ended at the end of 2020. In addition, all participants provided verbal informed consent forms, and all interviewers and supervisors participating in this survey wore identification issued by Peking University. Supervisors will conduct supervision, and all interviewers and supervisors have received strict professional training to understand the relevant content of the Statistics Law of the People’s Republic of China, Law of the People’s Republic of China on Guarding State Secrets, and Criminal Law of the People’s Republic of China, and strictly abide by relevant laws. All minors involved in the survey have obtained the consent of their parents or guardians.

Second, although the CFPS dataset is a longitudinal survey, children’s educational attainment probably remains unchanged once they have completed their schooling, and parents’ marital age is fixed. Consequently, once these key explanatory and dependent variables are determined, they remain stable. Using multiple waves of panel data could lead to repeated observations of the same individuals, such that, for instance, five observations of the same child would essentially contribute only one independent piece of information (As we found when matching the 2018 and 2020 CFPS samples, where approximately 90% of the observations were repeated). This could result in an overestimation of the estimated effects. Therefore, we opt not to use multi-wave panel data but instead conduct our empirical analysis based on a single wave of data. This is consistent with the findings of Ashok et al. (2024) and Saha et al. (2024) [47–48], who also emphasize the potential biases introduced by repeated observations in panel data and advocate for using single-wave data in similar empirical contexts.

The key research object of this study is the parents and their children. To obtain the one-to-one correspondence between children and parents, and to expand the age coverage of children, the database is processed as follows: ①According to the family relationship database provided by CFPS (2020), only those adult samples who have children and whose children are also surveyed in the questionnaire are saved to merge the parent database; ②Match the family information in the family database with the parent database to obtain detailed family characteristics; ③Merge the information of adults and their children, get the matched parent-child relationship database. Since this paper aims to investigate the impact of age at marriage on children’s education, only those children who completed studies are considered. For model (1), there are 4540 observed values of the processed child sample.

### 3.3 Key variables

The key explanatory variable is the parents’ marriage age, which uses the direct survey data provided by the CFPS (2020) database, “the date of marriage with the first spouse” minus the year of birth, to get the individual’s age at first marriage. The key variable explained is the education of children. This study adopts the children’s years of education measure, and we also used the completed education categories of children as a robustness test. Also, it should be noted that, considering some circumstances, a mother may have a first child at age 30 and a second at age 32. Compared to the distribution of the mother’s age at first birth, 30 may be “late”, but compared to the distribution of age at second birth, 32 may not be, so this article limits children to the first child in the family.

### 3.4 Control variables

The family is the environment that children first come into contact with, and the characteristics of both parents and family will affect their children’s education. To improve the validity and reliability of the results, and refer to existing research, this paper selects a series of variables reflecting the characteristics of parents and family as control variables. The variables of parental characteristics include parents’ education and health status, and the variables of family characteristics include per capita household income, number of children in the family, gender and age of the first child, urban and rural conditions, etc. Parents with higher education positively impact their children’s education outcomes [49]; parents with better health conditions will invest more energy in their children, while parental diseases hurt children’s education [50]. Berger and Fleisher (1984) [51] find that if the father or mother’s health in the family deteriorates, the other party will increase the market labor time or the care time for the patient, reducing the children’s energy input. The higher the income of the family, the more advantages including a suitable living environment and school conditions for children’s education will be provided [52]; when the internal resources of the family are limited, the number of children in the family is an essential factor for parents to invest in their children’s human capital [53]; the gender of children affects parents’ investment decisions [54]. Considering the typical urban-rural dual structure in China, this study also controls the variable of the location of the household.

The gender of the children is 1 for boys and 0 for girls. This article takes the natural logarithm of per capita household income. Parents’ education is measured by their parents’ years of education, and parents’ health status is measured by self-evaluated health, which is evaluated by the degree of health: 1 is very unhealthy, 2 is unhealthy, 3 is relatively unhealthy, 4 is fair, and 5 is very healthy. The family is assigned a value of 1 in the city, and the family is assigned a value of 0 in the countryside. Table 1 shows the descriptive statistical results of the main variables.

**Table 1:** Descriptive statistics of main variables.

Variable Name	Mean	S.D.	Minimum	Maximum
Education of children	4.157	1.336	1	8
Children’s years of education	12.071	3.482	0	22
Marriage age	22.936	3.400	16	55
The square of marriage age	5.376	1.770	2.560	30.250
**Parental characteristics**				
Age	57.164	8.010	29	91
Gender	0.479	0.500	0	1
Education	2.395	1.152	1	7
Health	2.804	1.274	1	5
**Family characteristics**				
Income	9.826	1.001	0	13.564
Number of children	1.976	0.920	1	7
Child gender	0.683	0.465	0	1
Child age	31.251	6.398	16	44
Urban and rural	0.568	0.495	0	1

What needs to be explained is that 68 percent of children in the samples are boys, which is different from the gender ratio at natural birth. For such an unbalanced gender structure of children in the sample, there are some explanations. The first reason is that for children who have completed their studies, their average age has reached 31 years old (as shown in Table 1), and most of them are starting a family and career. In China, the patriarchal society is dominant, with many girls marrying into their husbands and boys often staying at home to live with the previous generation. Therefore, in the questionnaire survey, the parents and their sons are usually the ones who can enter the questionnaire, which is reflected in the fact that males make up a more significant proportion of the children. Second, this article limits the sample to the first child in a family, as the traditional preference for sons over daughters can lead to some gender selection behaviors, which also increases the proportion of males.

Meanwhile, this article argues that more than 40 years after China began its reform, the Family Planning Policy (This policy is a population control policy implemented in China in 1978, mainly aimed at limiting the number of children to one. It is strictly enforced in cities, and in rural areas, if the first child is a girl, couples can also have a second child) and Education Expansion have increased women’s education. Chinese men have always had a higher level of education, in this case, a higher proportion of men is less likely to cause bias in the estimated results and may underestimate them.

### 3.5 Further descriptive analysis

To further examine the variations in key variables across different regions and genders, we conducted descriptive statistical analyses by categorizing the data into subgroups, such as urban versus rural areas and parental gender. The results are presented in Table 2 (Considering the length limitations, we have included the detailed descriptive statistics tables by parental gender and urban-rural classification in Appendix A). On average, rural individuals and females tend to marry at a younger age, with a mean of approximately 22 years. In contrast, urban individuals and males marry later, with a mean of around 23 years. This seemingly identical mean value reflects underlying distributional differences that merit further exploration. Additionally, a significant urban-rural gap exists in children’s educational attainment due to disparities in educational resources, parental emphasis on education, and other socioeconomic factors. On average, urban children receive approximately three more years of schooling than their rural counterparts.

**Table 2:** Descriptive statistics of key variables by region and parental gender (Mean value).

Variable Name	Sum (3980)	Rural (N = 1721)	Urban (N = 2259)	Mother (N = 2075)	Father (N = 1905)
Education of children	4.157	3.604	4.579	4.172	4.142
Children’s years of education	12.071	10.662	13.144	12.112	12.027
Marriage age	22.936	22.392	23.351	22.180	23.761
The square of marriage age	5.376	5.123	5.570	5.014	5.771

Moreover, this study examines the human capital outcomes of children whose parents married at different age ranges. As shown in the descriptive statistics in Table 3, children’s educational attainment exhibits a non-linear pattern concerning parental marriage age—it first increases and then declines rather than following a simple linear relationship. Specifically, starting from a parental marriage age of 28, children’s educational attainment begins to decrease. However, relying solely on descriptive statistics to infer an inverted U-shaped relationship between the dependent and key explanatory variables is not methodologically robust. Therefore, in Chapter 4, we introduce a squared term to test this relationship formally. Additionally, Table 3 reveals that the most significant proportion of parents married between the ages of 20 and 23, accounting for approximately 50% of the total sample. This highlights a notable discrepancy between the average marriage age of the parental cohort under study—whose children have completed their education—and the significantly delayed average marriage age observed in the current generation.

**Table 3:** Descriptive statistics of children’s human capital by parental marriage age.

Marriage age classification	Mean value of children’s education	Observations	Proportion
16-19 years old	10.621	485	12.186%
20-23 years old	12.109	2031	51.030%
24-27 years old	12.676	1149	28.869%
28-31 years old	11.953	233	5.854%
Over 32	11.573	82	2.060%
Sum	12.071	3980	100%

## 4. Results

### 4.1 The impact of parents’ marriage age on children’s educational human capital

#### 4.1.1 Benchmark regression results.

Table 4 reports the estimated effects of parental marriage age on their children’s education outcomes. OLS regression results from columns (1) and (2) show that the parents’ late marriage significantly impacts their children’s education (The first column is the category of children’s education, with a value range of 1–8. The second column is the length of education of the children, with a value range of 0–22. The same is as below). However, when parents marry later than a certain age, this positive effect will turn negative. This means there is an inverted U-shaped relationship between the marriage age of parents and the accumulation of human capital in children’s education. Further calculations have shown that, on the one hand, when parents married one year later, their children’s average years of schooling increased by 0.29 years, and about 2.35% (the average age of children in the sample is 12.22 years). This article calculates through a formula that the marriage age at the turning point is approximately 29.49 years old, which means when parents get married before the age of 29.49, getting married later is more beneficial for their children’s human capital; When parents get married beyond the age, getting married later may harm their children’s acquisition of education.

**Table 4:** Regression results of parents’ marriage age on children’s education.

Variables	OLS		Oprobit		OLS	
	(1)	(2)	(3)	(4)	(5)	(6)
Marriage age	0.110^***^ (0.041)	0.297^**^ (0.117)	0.107^***^ (0.040)	0.107^***^ (0.040)	0.139^***^ (0.038)	0.365^***^ (0.108)
The square of marriage age	-0.190^**^ (0.076)	-0.503^**^ (0.219)	-0.185^**^ (0.075)	-0.184^**^ (0.075)	-0.243^***^ (0.074)	-0.635^***^ (0.208)
Age	-0.008 (0.007)	-0.034^*^ (0.019)	-0.008 (0.007)	-0.009 (0.007)	-0.013^**^ (0.005)	-0.036^**^ (0.015)
Gender	-0.145^***^ (0.024)	-0.351^***^ (0.064)	-0.139^***^ (0.024)	-0.136^***^ (0.024)	-0.073^***^ (0.020)	-0.231^***^ (0.053)
Education	0.257^***^ (0.021)	0.618^***^ (0.054)	0.251^***^ (0.020)	0.251^***^ (0.020)	0.148^***^ (0.018)	0.388^***^ (0.046)
Health	-0.019 (0.016)	-0.064 (0.043)	-0.021 (0.015)	-0.024 (0.015)	-0.015 (0.013)	-0.059^*^ (0.034)
Income	0.240^***^ (0.042)	0.641^***^ (0.107)	0.233^***^ (0.042)	0.232^***^ (0.041)	0.237^***^ (0.036)	0.657^***^ (0.092)
Number of children	-0.121^***^ (0.031)	-0.292^***^ (0.086)	-0.116^***^ (0.031)	-0.114^***^ (0.031)	-0.109^***^ (0.023)	-0.247^***^ (0.066)
Child gender	-0.329^***^ (0.051)	-0.752^***^ (0.132)	-0.308^***^ (00.050)	-0.313^***^ (0.049)	-0.283^***^ (0.043)	-0.690^***^ (0.112)
Child age	-0.018^**^ (0.008)	-0.049^**^ (0.022)	-0.016^**^ (0.008)	-0.016^**^ (0.008)	0.006 (0.005)	-0.023 (0.016)
Urban and rural	0.587^***^ (0.058)	1.495^***^ (0.154)	0.547^***^ (0.057)	0.543^***^ (0.057)	0.478^***^ (0.049)	1.204^***^ (0.131)
Constant term	0.638 (0.688)	3.703^**^ (1.864)	—	—	0.020 (0.607)	2.609 (1.660)
Regional effects	control	control	control	control	control	control
N	3980	3980	3980	3980	5763	5763
R^2^	0.345	0.336	0.126	0.119	0.244	0.281

Note: (1)

***,

**,

*are significant at 1%, 5%, and 10% respectively; standard errors are in parentheses, the same is as below. (2) The column (2) is the benchmark regression.

The estimated coefficients of the control variables are consistent with existing research. Due to the scarcity of resources within the family, the greater the number of children, the lower the average educational investment the children receive and the lower the education years. The higher the family income, the longer the parent’s education, the higher the education outcomes of the children. An interesting finding is that the estimated coefficient for the gender of children is significantly negative, indicating that in recent years, the education level of girls has gradually improved, even surpassing that of boys. The possible reason is that with the implementation of the Family Planning Policy, when a family is limited to only one child, even if it is a daughter, parents will invest in their children’s human capital. Finally, compared to the children of rural families, the education outcomes of children in urban families are higher.

This article also changes the estimation model and the selection of research objects to demonstrate the robustness of the results. Considering the discrete characteristics of the education variable, this study draws on the ordered Probit model as a robustness test of OLS regression. The results are shown in columns (3) and (4) from Table 4, the significance and estimated parameter signs support the benchmark regression results. At the same time, parents’ higher education and higher family income increase children’s education probability.

Since the enrollment age for eligible children in China is 6 years old and the nine-year compulsory education system is implemented, eligible children enjoy nine years of basic free school education. After the age of 15, children will face three choices: not being able to attend high school and directly participating in work, vocational high school (with a term of 3 or 5 years, making it challenging to enter undergraduate universities), and ordinary high school (with a term of 3 years, a way to participating in the college entrance examination typically). The educational results under each choice are entirely different. Therefore, this article also replaces the limit of “completed education” in the benchmark regression with “15 years old and above (excluding 15 years old)”, and the regression results are shown in columns (5) and (6) of Table 4. The estimated coefficients confirm the robustness of the benchmark conclusion.

#### 4.1.2 Robustness tests.

① Introducing instrumental variables. Although children’s educational human capital does not affect the age at which their parents first marry, there may still be omissions of unobservable variables or self-selection, leading to endogeneity issues. For example, some individuals choose to delay marriage to provide their children with a better material foundation. To alleviate potential endogeneity issues, this article adopts “the closest distance from the province where the parents are located to the coastal port” (The data on the shortest distance from each province to the nearest coastal port came from PPData database) as an instrumental variable for the age at marriage of parents. The reason for selecting this variable is that being closer to coastal ports means more exposure to diverse cultures worldwide, accepting more concepts such as free love and independence at the marriage, and being more likely to choose to delay marriage [55]. Meanwhile, natural geographic variables have gained widespread recognition in research due to their homogeneity. Therefore, this variable can better meet the two conditions of correlation and homogeneity. From the second column of Table 5, it can be observed that the farther away from coastal ports, the earlier the age of marriage. The second-stage regression results in column (3) show that the positive effect of marriage age on children’s education is still significant.

**Table 5:** Estimation results of instrumental variables.

Variables	(1) Benchmark regression	(2) Marriage age	(3) Children’s education
Marriage age	0.297^**^ (0.117)		3.842^**^ (1.708)
The closest distance to coastal ports		-0.035^***^ (0.009)	
Control variables	control	control	control
N	3980	3980	3980
R^2^	0.336	0.972	—

② Group regression by turning point. The conclusion of Table 4 indicates an inverted U-shaped relationship between the accumulation of educational human capital of parents at marriage age and their children. But does the estimated turning point of the quadratic equation have economic significance? This article further divides the sample into two groups based on the calculated marriage age turning point of 29.49 that affects children’s education (since age is an integer, a value of 30 is taken here). The regression results are presented in columns (1) and (2) of Table 6. It is found that for couples married before the age of 30, the age of marriage significantly positively impacts their children’s education, and the coefficients are more significant than the estimated coefficients of the baseline regression. For couples who get married after age 30, the influence of marriage age turns negative, but it is not statistically significant. This may be because, for most eligible young people in China, relatively few get married after 30, with only 147 samples.
③ Group regression by parents’ age. Parents born in different years have different historical backgrounds. For example, couples born in the era of the Family Planning Policy are limited in the number of children they can have. When there is only one child in the family, all the capital in the family is invested in that child, and their educational human capital is also higher. Therefore, this article divides parents into three categories based on their birth years: “1950-1960”, “1960-1970”, and “1970-1980” (due to the small sample of parents born after 1980, only 12 were included in the regression, so this group was discarded) and grouped for regression. The results of OLS regression after the regroup in columns (3) to (5) of Table 6 show that after changing the grouping standard, the estimated coefficient of marriage age on children’s education is positive at a significant level of 1%. The extension of the marriage age significantly improved the education of the children. In summary, the conclusion that late marriage improves children’s education is robust.

**Table 6:** Estimation results of group regression.

Variables	(1) Under 30	(2) Over 30	(3) Before 1960	(4) 1960-1970	(5) 1970-1980
Marriage age	1.295^***^ (0.262)	-0.089 (0.641)	0.159 (0.127)	0.972^***^ (0.236)	2.436^***^ (0.577)
The square of marriage age	-2.671^***^ (0.568)	0.135 (0.788)	-0.258 (0.214)	-1.840^***^ (0.471)	-5.196^***^ (1.292)
Control variables	control	control	control	control	control
Regional effects	control	control	control	control	control
N	3833	147	1449	1827	692
R^2^	0.339	0.547	0.356	0.351	0.325

### 4.2 The different effects of marriage age between fathers and mothers on children’s educational human capital

#### 4.2.1 Benchmark regression results.

Existing studies have shown that mothers spend more time caring and educating for their children than fathers [42]. Then, is there a difference in the influence of fathers and mothers on their children’s education? The article next examines the impact of the marriage age of fathers and mothers on the education outcomes of their children to test Hypothesis 2. From the results reported in columns (1) and (4) of Table 7, it can be found that just the marriage age of the mother can significantly improve the education outcomes of the children. Also, we adopted the SUEST, testing based on a Seemingly Unrelated Regression (SUR), to statistically test whether there is a significant difference between the two coefficients. The P-values of the estimated coefficients for marriage age and the square term of marriage age are 0.012 and 0.015, respectively, indicating that the difference between fathers and mothers is statistically significant. The results based on the ordered probit model of columns (2) and (5) confirm the robustness of the results. Further regress and perform SUR tests on parents whose first marriage age is before 30. It was found that although the estimated coefficient of 1.615 for the mother’s marriage age is slightly higher than the estimated coefficient of 1.114 for the father’s marriage age, it is not statistically significant (The P-values of the estimated coefficients for marriage age and the square term of marriage age are 0.374 and 0.387).

**Table 7:** Regression results of parents’ marriage age and children’s education.

Variables	Mother			Father		
	(1) OLS	(2) Oprobit	(3) Under 30	(4) OLS	(5) Oprobit	(6) Under 30
Marriage age	0.595^***^ (0.167)	0.218^***^ (0.061)	1.615^***^ (0.318)	0.092 (0.129)	0.037 (0.042)	1.114^***^ (0.392)
The square of marriage age	-1.087^***^ (0.335)	-0.405^***^ (0.123)	-3.368^***^ (0.700)	-0.127 (0.233)	-0.055 (0.076)	-2.297^***^ (0.840)
Control variables	control	control	control	control	control	control
Regional effects	control	control	control	control	control	control
N	2075	2075	2021	1905	1905	1812
R^2^	0.345	0.124	0.343	0.334	0.116	0.342

#### 4.2.2 Considering changes in historical context and gender roles.

Over different historical periods, shifts in the availability of educational resources, societal attitudes toward marriage, and family structures have influenced the effect of parental marital age on children’s education. So, have parental characteristics changed over time and subsequently influenced children’s education? As female educational attainment has improved and labor force participation has risen, the role of mothers in children’s education has evolved across different periods. In earlier historical contexts, mothers primarily assumed caregiving responsibilities within the household. However, as modern women engage more actively in the workforce, their support for children’s education may have shifted from direct time investment to financial contributions or access to educational resources. To further examine whether the effect of parental marital age varies across historical periods, we conducted an in-depth analysis from two perspectives:

First, according to Section 4.1.2, we performed subgroup regression analysis based on parental birth cohorts. We categorized parents into three cohorts (born before 1960, between 1960 and 1970, and between 1970 and 1980) to investigate whether differences in historical contexts led to variations in how parental marital age influences children’s education. The empirical results in Table 8 show that the inverted U-shaped relationship between maternal marital age and children’s education is significant across all cohorts, with the peak effect consistently observed between ages 24 and 27. This indicates that the impact pattern of maternal marital age on children’s education remains stable across generations. In contrast, the effect of paternal marital age is insignificant for fathers born before 1960. Still, it becomes significant for those born between 1960 and 1970 and between 1970 and 1980, with a stable peak effect between ages 22 and 27. These findings suggest two key insights. First, while parental characteristics may have evolved, the fundamental influence of marital age on children’s education has not undergone systematic distortions. Second, consistent with our baseline results, the educational benefits associated with maternal marital age are more pronounced than those of paternal marital age.

**Table 8:** Parental differences (Subgroup regression results by parental birth cohorts).

Variables	Mother			Father		
	(1) Before 1960	(2) 1960-1970	(3) 1970-1980	(4) Before 1960	(5) 1960-1970	(6) 1970-1980
Marriage age	0.340^**^ (0.156)	0.925^**^ (0.381)	2.060^***^ (0.678)	0.111 (0.146)	1.005^***^ (0.255)	2.751^***^ (0.967)
The square of marriage age	-0.645^**^ (0.266)	-1.776^**^ (0.805)	-4.166^***^ (1.492)	-0.155 (0.237)	-1.863^***^ (0.485)	-6.126^***^ (2.137)
Control variables	control	control	control	control	control	control
Regional effects	control	control	control	control	control	control
N	683	985	400	766	842	292
R^2^	0.370	0.366	0.338	0.365	0.350	0.374
Turning point	26.378	26.034	24.727	—	26.980	22.451

Second, we conducted a period-based regression analysis based on parental marriage timing. Given the variations in marriage patterns across different historical periods in China, we categorized parents into two groups based on their marriage timing to examine whether the effect of marital age on children’s education has adjusted over time. Specifically, we divided marriage timing into two periods: before 1980 and 1980 or later, and performed separate regressions for each. This classification is primarily based on enacting the new marriage law in 1980, which introduced revised legal marriage age requirements. Before 1981, China adhered to the 1970s’ late marriage and late childbirth policy, resulting in relatively high average marriage ages. However, after 1981, the legal marriage age was set at 22 for men and 20 for women, leading to a decline in marriage age. Accordingly, we used this historical policy shift for our subgroup regression analysis.

The empirical results presented in Table 9 indicate that the effect of maternal marital age on children’s education remains significant before and after 1980, consistently exhibiting an inverted U-shaped relationship. This suggests that regardless of whether a mother married before or after 1980, both early and late marriages negatively affect children’s educational outcomes. In contrast, marriage at an optimal age yields the most positive impact. This finding may be attributed to the primary caregiving role of mothers within the family—despite changes in the broader social environment, a mother’s direct involvement in early childhood education (such as time spent with children and participation in early learning activities) remains a crucial determinant of educational success. Additionally, since the biological window for childbirth is relatively stable, an appropriately timed marriage is more likely to allow mothers to give birth within an optimal reproductive age, benefiting both children’s physical development and educational investment.

**Table 9:** Parental differences (Subgroup regression results by parental marriage timing).

Variables	Mother		Father	
	(1) Before 1980	(2) After 1980	(3) Before 1980	(4) After 1980
Marriage age	1.157^**^ (0.517)	0.559^***^ (0.172)	1.019^***^ (0.358)	0.067 (0.128)
The square of marriage age	-2.373^**^ (1.127)	-1.017^***^ (0.322)	-2.088^***^ (0.708)	-0.011 (0.224)
Control variables	control	control	control	control
Regional effects	control	control	control	control
N	507	1568	472	1433
R^2^	0.315	0.325	0.340	0.316
Turning point	24.379	27.490	24.398	—

In contrast, the effect of paternal marital age remains significant for fathers who married before 1980, following an inverted U-shaped pattern. However, for those who married after 1980, while the estimated coefficients maintain a similar direction, the overall effect is no longer statistically significant. This shift may be attributed to the relatively delayed role of fathers in family education and their greater susceptibility to societal changes. Compared to mothers, fathers tend to have less direct involvement in children’s education, with their contributions primarily reflected in economic support and the accumulation of social capital. As female educational attainment increased and female labor force participation rose in the post-1980 era, family caregiving dynamics underwent significant transformations. With mothers contributing more economically, the influence of paternal marital age on children’s education may have diminished as a key determinant.

Overall, these findings suggest that in the context of post-1980 social transformations, the influence of maternal marital age on children’s education has remained robust. In contrast, the effect of paternal marital age has weakened due to socioeconomic development and shifts in family caregiving structures. This further supports the notion that social changes shape family decision-making and intergenerational transmission mechanisms, highlighting the need to consider gender differences and their evolution across historical periods when examining the effects of marital age.

## 5. Conclusion and discussion

This article uses CFPS data to empirically investigate the impact of parents’ marriage age on their children’s education. The study found that: There is an inverted U-shaped relationship between the marriage age of parents and the accumulation of human capital in children’s education. This conclusion is still significantly robust after changing the estimation method, choosing different children, and using the IV method to improve the endogenous problem. Furthermore, the impact of parents’ marriage age on their children’s education is different between fathers and mothers. Mothers have a more significant impact on their children’s education than fathers. However, this conclusion is not statistically significant for couples who get married before 30.

The core conclusion of this study—that moderate delays in marriage contribute to the enhancement of children’s educational human capital—is consistent with a large body of existing research [2,5,6,44,47,56–58]. However, our primary contribution is identifying the nonlinear relationship between parental marriage age and children’s long-term educational attainment.

Existing studies have primarily focused on two aspects. First, early marriage remains prevalent in many low- and middle-income countries or contexts with significant gender disparities. Prior research has widely documented the adverse effects of child marriage and early marriage, highlighting that delaying marriage enhances maternal education, improves household economic conditions, and ultimately fosters more significant human capital accumulation in children (Field & Ambrus, 2008; Saha et al., 2024). Consequently, much of the literature has concentrated on strategies to mitigate early marriage in countries such as India [5], Uganda [6], and Pakistan [47], providing policy recommendations tailored to these contexts.

Second, existing research has primarily examined the short-term academic outcomes of children, such as literacy, cognitive test performance, and mathematics proficiency [5,58]. However, these studies often overlook the long-term impact of parental marriage age on children’s educational attainment. Specifically, they do not address whether progressively delaying marriage consistently yields positive effects or an optimal marriage age range exists.

The marriage age has recently risen significantly in some developing countries, such as China. According to the Seventh National Population Census of China, the average age at first marriage reached 28.67 years in 2020 (29.38 years for men and 27.95 years for women). Does this trend necessarily lead to positive educational outcomes for children? From a life-cycle perspective, individuals and families face different resource allocation constraints and decision-making priorities at various developmental stages. For example, early marriage may result in insufficient economic capital accumulation, whereas excessively delayed marriage could limit parents’ time investment and energy devoted to their children [28]. Therefore, this study explores whether prolonged delays in marriage consistently benefit children and investigates the potential nonlinear relationship between parental marriage age and children’s educational attainment, particularly when considering their final educational achievements in adulthood.

One of the most directly relevant studies is Fall et al. (2015) [59], which utilized cohort data to examine the association between maternal marriage age and children’s long-term developmental outcomes. Their findings suggest that children born to young mothers (<19 years) and older mothers (>35 years) face heightened risks in both health and education. However, their study was primarily descriptive, focusing on correlations rather than providing rigorous causal identification or theoretical mechanisms.

Additionally, while prior studies have examined heterogeneity across different dimensions—such as the effects of parental marriage age on children’s health and education [5] and gender-based disparities [47]—they have paid little attention to the differential effects of paternal and maternal marriage ages. Although Ashok et al. (2024) [47] found that fathers’ marriage age is positively associated with daughters’ school enrollment and primary school completion rates, they did not explicitly compare the effects of paternal versus maternal marriage age.

Overall, leveraging micro-level household data from China, this study systematically investigates the inverse U-shaped relationship between parental marriage age and children’s educational human capital, making three key theoretical contributions: (1) it highlights the long-term effects of parental marriage age on children’s human capital accumulation, expanding the research frontiers of family and education economics; (2) it empirically validates the nonlinear relationship between marriage age and children’s educational attainment, challenging traditional linear assumptions and providing more nuanced policy implications; and (3) it identifies a significantly more substantial impact of maternal marriage age compared to paternal marriage age, offering new insights into intra-household resource allocation and intergenerational transmission mechanisms.

At the practical level, although marital status is the result of parents’ self-selection, we propose the following policy recommendations to promote family stability and enhance parental investment in children’s education.

(1) Promoting Rational Perspectives on Marriage and Encouraging Timely Marital Decisions. As societies modernize, the average age at marriage has been rising among young adults. Existing research highlights the “timing effect” and “level effect” of delayed marriage on fertility, while this study further provides empirical evidence suggesting that excessive delays in marriage may weaken parental support for children’s education. Therefore, governments and social institutions should leverage media and public education platforms to promote rational perspectives on marriage and encourage young people to make informed marital decisions at an appropriate age. Additionally, services such as relationship counseling and psychological support should be made available to help individuals navigate real-life challenges, thereby mitigating the potential negative impact of marital issues on children’s educational outcomes.
(2) Alleviating Economic Pressures to Enhance the Educational Environment for Children. Late marriage is often associated with financial concerns about future family life, particularly child-rearing costs. To address this, governments should implement economic policies such as marriage-related financial subsidies, tax incentives, and housing assistance to alleviate the economic burden on individuals of marriageable age. These measures can contribute to a more stable family environment, ultimately fostering better educational conditions for children.
(3) Providing Targeted Support to Enhance Parental Investment in Education. Early-Marriage Parents: Expand access to educational resources by offering parenting courses and family education consultations to strengthen their capacity to invest in children’s education. Parents Who Marry at an Appropriate Age: Provide childcare support to help them balance work and family responsibilities, ensuring a conducive environment for children’s development. Late-Marriage Parents: Encourage lifelong learning to enhance their educational attainment, setting a positive example for their children and fostering a culture of continuous learning within the family.
(4) Enhancing Gender-Sensitive Policies to Promote Paternal Involvement in Childcare. Encouraging greater paternal involvement in child-rearing is crucial for children’s development. Governments should introduce policies such as paid paternity leave to facilitate a more equitable division of parenting responsibilities within households, strengthening fathers’ active participation in their children’s upbringing.

However, there may still be some shortcomings in this article, such as limited sample size, limited sample size for marriage after the age of 30 in group regression, and the mechanism of the influence of parental marriage age on children’s education has not been validated. In the future, as the age of first marriage continues to increase, obtaining more data can further focus on the impact on the education of children for couples who marry after the age of 30. Pay more attention to testing the mechanisms of the inverted U-shaped effect of parental marriage age on children’s human capital accumulation.

## Supporting information

S1 Appendix Descriptive statistics of key variables by region and parental gender. Appendix A presents descriptive statistics of key variables disaggregated by region (urban vs. rural) and parental gender (mother vs. father). These tables are intended to illustrate sample heterogeneity and contextual differences in the effects of parental marriage age on children’s educational outcomes. Table A.1 compares urban and rural families, showing disparities in marriage age, educational attainment, income, and family structure. Table A.2 contrasts maternal and paternal characteristics, highlighting gender-based differences in marriage timing, education, and health. These data support the robustness of the main analysis by emphasizing socio-demographic variability.
(DOCX)
