# Supplementary material for: The long-term effects of parental marriage age on children’s educational human capital
Source: PLoS One. 2025 May 7;20(5):e0322151. doi: 10.1371/journal.pone.0322151 (PMC12057889; doi:10.1371/journal.pone.0322151)
Supplement: S1 Appendix — Descriptive statistics of key variables by region and parental gender. Appendix A presents descriptive statistics of key variables disaggregated by region (urban vs. rural) and parental gender (mother vs. father). These tables are intended to illustrate sample heterogeneity and contextual differences in the effects of parental marriage age on children’s educational outcomes. Table A.1 compares urban and rural families, showing disparities in marriage age, educational attainment, income, and family structure. Table A.2 contrasts maternal and paternal characteristics, highlighting gender-based differences in marriage timing, education, and health. These data support the robustness of the main analysis by emphasizing socio-demographic variability. (DOCX) [file pone.0322151.s001.docx]

**Appendix A. Descriptive statistics of key variables by region and parental gender**

We conducted descriptive statistical analyses of key variables separately based on parental gender and urban-rural differences to examine potential heterogeneity in the effects of parental marriage age on children’s educational human capital. Table A.1 presents the descriptive statistics by urban and rural regions, highlighting differences in children’s education, parental characteristics, and family characteristics. Table A.2 presents the descriptive statistics by parental gender, facilitating a comparison between mothers and fathers. These analyses provide a comprehensive overview of the sample characteristics, emphasizing the importance of considering contextual factors in understanding the long-term effects of parental marriage age.

Specifically, the descriptive statistics in Table A.1 reveal differences between urban and rural areas in key variables. Urban children tend to have higher educational levels and longer years of education compared to their rural counterparts. Parents in urban areas marry later (23.351 years vs. 22.392 years), indicating a tendency toward delayed marriage. Urban parents are generally better educated and have a higher average income, with fewer children, reflecting the influence of urbanization on family structure. Health and age differences are minimal. In Table A.2, the gender-based analysis reveals that mothers marry at a younger age (22.180 years) than fathers (23.761 years), while fathers have a slight advantage in education and health status. These results suggest that urbanization and gender roles have a significant influence on educational and economic outcomes, highlighting the need for further research on the mechanisms underlying these disparities.

Table A.1 Descriptive statistics of key variables by region

| **Variable Name** | Rural（N=1721） | | Urban（N=2259） | |
| --- | --- | --- | --- | --- |
|  | Mean | S.D. | Mean | S.D. |
| Education of children | 3.604 | 1.174 | 4.579 | 1.297 |
| Children’s years of education | 10.662 | 3.250 | 13.144 | 3.263 |
| Marriage age | 22.392 | 3.298 | 23.351 | 3.418 |
| The square of marriage age | 5.123 | 1.670 | 5.570 | 1.819 |
| **Parental characteristics** |  |  |  |  |
| Age | 57.144 | 8.301 | 57.179 | 7.783 |
| Gender | 0.491 | 0.500 | 0.469 | 0.499 |
| Education | 2.061 | 0.979 | 2.649 | 1.208 |
| Health | 2.793 | 1.320 | 2.811 | 1.238 |
| **Family characteristics** |  |  |  |  |
| Income | 9.576 | 0.831 | 10.016 | 1.075 |
| Number of children | 2.158 | 0.918 | 1.838 | 0.897 |
| Child gender | 0.756 | 0.430 | 0.628 | 0.483 |
| Child age | 31.353 | 6.521 | 31.172 | 6.303 |

Table A.2 Descriptive statistics of key variables by parental gender

| **Variable Name** | Mother（N=2075） | | Father（N=1905） | |
| --- | --- | --- | --- | --- |
|  | Mean | S.D. | Mean | S.D. |
| Education of children | 4.172 | 1.327 | 4.142 | 1.345 |
| Children’s years of education | 12.112 | 3.451 | 12.027 | 3.514 |
| Marriage age | 22.180 | 3.076 | 23.761 | 3.542 |
| The square of marriage age | 5.014 | 1.527 | 5.771 | 1.925 |
| **Parental characteristics** |  |  |  |  |
| Age | 56.477 | 7.906 | 57.912 | 8.057 |
| Education | 2.189 | 1.150 | 2.618 | 1.113 |
| Health | 2.696 | 1.276 | 2.921 | 1.263 |
| **Family characteristics** |  |  |  |  |
| Income | 9.825 | 0.997 | 9.827 | 1.006 |
| Number of children | 1.974 | 0.921 | 1.978 | 0.919 |
| Child gender | 0.680 | 0.466 | 0.687 | 0.464 |
| Child age | 31.327 | 6.399 | 31.167 | 6.397 |
| Urban and rural | 0.578 | 0.494 | 0.556 | 0.497 |
